# Supplementary material for: Impact of cancer-associated mutations in Hsh155/SF3b1 HEAT repeats 9-12 on pre-mRNA splicing in Saccharomyces cerevisiae
Source: PLoS One. 2020 Apr 22;15(4):e0229315. doi: 10.1371/journal.pone.0229315 (PMC7176370; doi:10.1371/journal.pone.0229315)
Supplement: S1 Raw images — (PDF) [file pone.0229315.s001.pdf]

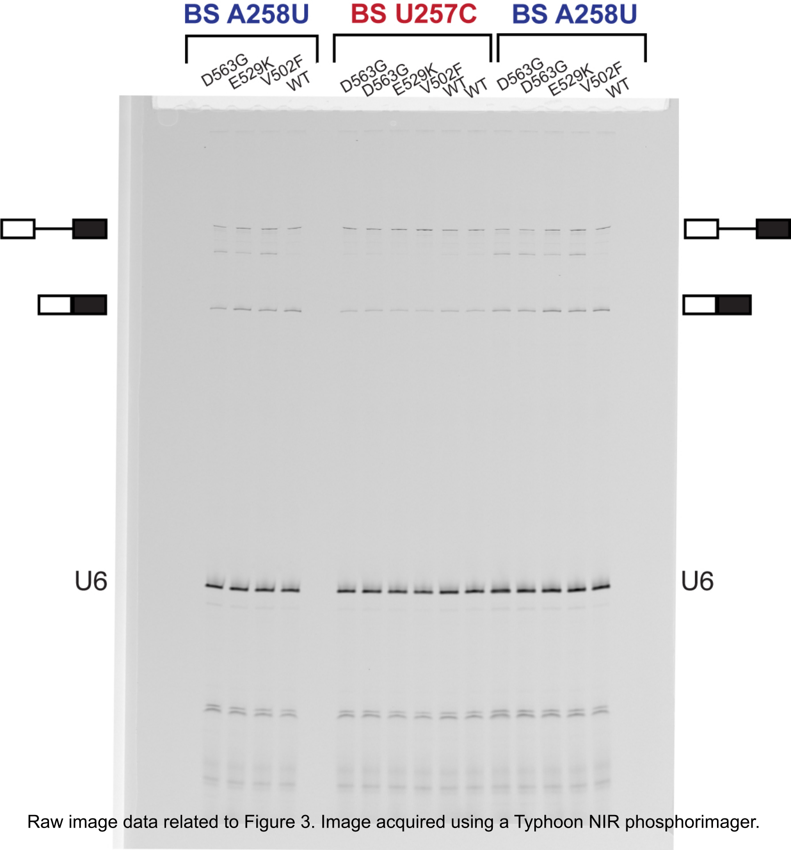

Raw image data related to Figure 3. Image acquired using a Typhoon NIR phosphorimager.

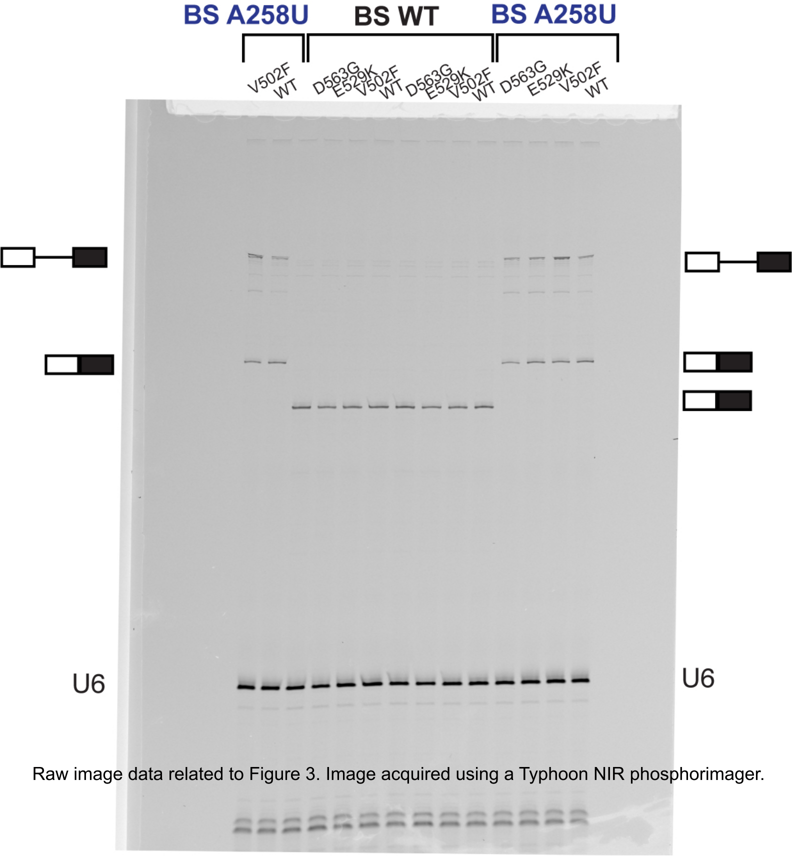

Raw image data related to Figure 3. Image acquired using a Typhoon NIR phosphorimager.
