# Supplementary material for: Impact of cancer-associated mutations in Hsh155/SF3b1 HEAT repeats 9-12 on pre-mRNA splicing in Saccharomyces cerevisiae
Source: PLoS One. 2020 Apr 22;15(4):e0229315. doi: 10.1371/journal.pone.0229315 (PMC7176370; doi:10.1371/journal.pone.0229315)
Supplement: S1 Table — (PDF) [file pone.0229315.s002.pdf]

S1 Table. Yeast strains used in this study.

Name	Genotype	Description
yAAH0418	MATa prc1407 prb11122 pep43 leu2 trp1 ura352 gal2, hsh155::KanMx, pRS416- Hsh155^{WT}	Strain used to generate HSH155 mutants by plasmid shuffle.
yAAH0424	MATa prc1407 prb11122 pep43 leu2 trp1 ura352 gal2, hsh155::KanMx, pRS414-Hsh155^{WT}	Strain expressing Hsh155^{WT}. Used for temperature assay.
yAAH2151	MATa prc1407 prb11122 pep43 leu2 trp1 ura352 gal2, hsh155::KanMx, pRS414-Hsh155^{V502F}	Strain expressing Hsh155^{V502F}. Used for temperature assay.
yAAH1971	MATa prc1407 prb11122 pep43 leu2 trp1 ura352 gal2, hsh155::KanMx, pRS414-Hsh155^{E529K}	Strain expressing Hsh155^{E529K}. Used for temperature assay.
yAAH2152	MATa prc1407 prb11122 pep43 leu2 trp1 ura352 gal2, hsh155::KanMx, pRS414-Hsh155^{E531K}	Strain expressing Hsh155^{E531K}. Used for temperature assay.
yAAH1922	MATa prc1407 prb11122 pep43 leu2 trp1 ura352 gal2, hsh155::KanMx, pRS414-Hsh155^{D563G}	Strain expressing Hsh155^{D563G}. Used for temperature assay.
yAAH2149	MATa prc1407 prb11122 pep43 leu2 trp1 ura352 gal2, hsh155::KanMx, pRS414-Hsh155^{E571G}	Strain expressing Hsh155^{E571G}. Used for temperature assay.
yAAH2150	MATa prc1407 prb11122 pep43 leu2 trp1 ura352 gal2, hsh155::KanMx, pRS414-Hsh155^{E571K}	Strain expressing Hsh155^{E571K}. Used for temperature assay.
yAAH2153	MATa prc1407 prb11122 pep43 leu2 trp1 ura352 gal2, hsh155::KanMx, pRS414-Hsh155^{I626Q}	Strain expressing Hsh155^{I626Q}. Used for temperature assay.
yAAH0465	MATα cup1Δ ura3 his3 trp1 lys2 ade2 leu2 Hsh155::KanMx, pRS416-Hsh155^{WT}	Copper sensitive strain used to generate HSH155 mutants for ACT1-CUP1 by plasmid shuffle. Hsh155 was deleted using KanMax cassette.
yAAH0648	MATα cup1Δ ura3 his3 trp1 lys2 ade2 leu2 Hsh155::KanMx, pRS414-Hsh155^{WT}	Copper sensitive strain expressing Hsh155^{WT}. Used for ACT1-CUP1 assay after transformation with reporter plasmids.
yAAH2156	MATα cup1Δ ura3 his3 trp1 lys2 ade2 leu2 Hsh155::KanMx, pRS414-Hsh155^{V502F}	Copper sensitive strain expressing Hsh155^{V502F}. Used for ACT1-CUP1 assay after transformation with reporter plasmids.
yAAH1999	MATα cup1Δ ura3 his3 trp1 lys2 ade2 leu2 Hsh155::KanMx, pRS414-Hsh155^{E529K}	Copper sensitive strain expressing Hsh155^{E529K}. Used for ACT1-CUP1 assay after transformation with reporter plasmids.
yAAH2157	MATα cup1Δ ura3 his3 trp1 lys2 ade2 leu2 Hsh155::KanMx, pRS414-Hsh155^{E531K}	Copper sensitive strain expressing Hsh155^{E531K}. Used for ACT1-CUP1 assay after transformation with reporter plasmids.
yAAH1921	MATα cup1Δ ura3 his3 trp1 lys2 ade2 leu2 Hsh155::KanMx, pRS414-Hsh155^{D563G}	Copper sensitive strain expressing Hsh155^{D563G}. Used for ACT1-CUP1 assay after transformation with reporter plasmids.
yAAH2154	MATα cup1Δ ura3 his3 trp1 lys2 ade2 leu2 Hsh155::KanMx, pRS414-Hsh155^{E571G}	Copper sensitive strain expressing Hsh155^{E571G}. Used for ACT1-CUP1 assay after transformation with reporter plasmids.
yAAH2155	MATα cup1Δ ura3 his3 trp1 lys2 ade2 leu2 Hsh155::KanMx, pRS414-Hsh155^{E571K}	Copper sensitive strain expressing Hsh155^{E571K}. Used for ACT1-CUP1 assay after transformation with reporter plasmids.

yAAH2158	MATα cup1Δ ura3 his3 trp1 lys2 ade2 leu2 Hsh155::KanMx, pRS414-Hsh155^{I626Q}	Copper sensitive strain expressing Hsh155^{I626Q}. Used for ACT1-CUP1 assay after transformation with reporter plasmids.
yAAH0650	MATα cup1Δ ura3 his3 trp1 lys2 ade2 leu2 Hsh155::KanMx, pRS414-Hsh155^{H331D}	Copper sensitive strain expressing Hsh155^{H331D}. Used for ACT1-CUP1 assay after transformation with reporter plasmids.
yAAH0635	MATα cup1Δ ura3 his3 trp1 lys2 ade2 leu2 Hsh155::KanMx, pRS414-Hsh155^{D450G}	Copper sensitive strain expressing Hsh155^{D450G}. Used for ACT1-CUP1 assay after transformation with reporter plasmids.
yTJC0298	MATα cup1Δ ura3 his3 trp1 lys2 ade2 leu2 Hsh155::KanMx, pRS414-Hsh155^{K740R}	Copper sensitive strain expressing Hsh155^{K740R}. Used for ACT1-CUP1 assay after transformation with reporter plasmids.
yTJC0300	MATα cup1Δ ura3 his3 trp1 lys2 ade2 leu2 Hsh155::KanMx, pRS414-Hsh155^{N747A}	Copper sensitive strain expressing Hsh155^{N747A}. Used for ACT1-CUP1 assay after transformation with reporter plasmids.
yAAH2237	MATα cup1Δ ura3 his3 trp1 lys2 ade2 leu2 Hsh155::KanMx, pRS414-Hsh155$^{H331D/V502F}$	Copper sensitive strain expressing Hsh155$^{H331D/V502F}$. Used for ACT1-CUP1 assay after transformation with reporter plasmids.
yAAH2236	MATα cup1Δ ura3 his3 trp1 lys2 ade2 leu2 Hsh155::KanMx, pRS414-Hsh155$^{D450G/V502F}$	Copper sensitive strain expressing Hsh155$^{D450G/V502F}$. Used for ACT1-CUP1 assay after transformation with reporter plasmids.
yAAH2238	MATα cup1Δ ura3 his3 trp1 lys2 ade2 leu2 Hsh155::KanMx, pRS414-Hsh155$^{K740R/V502F}$	Copper sensitive strain expressing Hsh155$^{K740R/V502F}$. Used for ACT1-CUP1 assay after transformation with reporter plasmids.
yAAH2239	MATα cup1Δ ura3 his3 trp1 lys2 ade2 leu2 Hsh155::KanMx, pRS414-Hsh155$^{N747A/V502F}$	Copper sensitive strain expressing Hsh155$^{N747A/V502F}$. Used for ACT1-CUP1 assay after transformation with reporter plasmids.
yAAH2214	MATα cup1Δ ura3 his3 trp1 lys2 ade2 leu2 Hsh155::KanMx, pRS416-Hsh155^{WT} prp2::hphMX pRS415-Prp2^{WT}	Used to generate Hsh155 mutants strains by plasmid shuffle. Prp2 was deleted using an hphMX deletion cassette.
yAAH2214	MATα cup1Δ ura3 his3 trp1 lys2 ade2 leu2 Hsh155::KanMx, pRS416-Hsh155^{WT} prp2::hphMX pRS415-Prp2^{Q548N}	Used to generate Hsh155 alleles by plasmid shuffle. Prp2 was deleted using an hphMX deletion cassette.
yAAH1915	MATα cup1Δ ura3 his3 trp1 lys2 ade2 leu2 Hsh155::KanMx, pRS414-Hsh155^{WT} prp2::hphMX pRS415-Prp2^{WT}	Copper sensitive strain expressing Hsh155^{WT} and Prp2^{WT} from plasmids. Used for temperature assays.
yAAH2221	MATα cup1Δ ura3 his3 trp1 lys2 ade2 leu2 Hsh155::KanMx, pRS414-Hsh155^{V502F} prp2::hphMX pRS415-Prp2^{WT}	Copper sensitive strain expressing Hsh155^{V502F} and Prp2^{WT} from plasmids. Used for temperature assays.
yAAH2222	MATα cup1Δ ura3 his3 trp1 lys2 ade2 leu2 Hsh155::KanMx, pRS414-Hsh155^{E529K} prp2::hphMX pRS415-Prp2^{WT}	Copper sensitive strain expressing Hsh155^{E529K} and Prp2^{WT} from plasmids. Used for temperature assays.
yAAH2219	MATα cup1Δ ura3 his3 trp1 lys2 ade2 leu2 Hsh155::KanMx, pRS414-Hsh155^{E531K} prp2::hphMX pRS415-Prp2^{WT}	Copper sensitive strain expressing Hsh155^{E531K} and Prp2^{WT} from plasmids. Used for temperature assays.
yAAH1964	MATα cup1Δ ura3 his3 trp1 lys2 ade2 leu2	Copper sensitive strain expressing Hsh155^{D563G} and Prp2^{WT} from plasmids. Used for temperature assays.

	Hsh155::KanMx, pRS414-Hsh155^{D563G} prp2::hphMX pRS415-Prp2^{WT}	
yAAH2218	MATα cup1Δ ura3 his3 trp1 lys2 ade2 leu2 Hsh155::KanMx, pRS414-Hsh155^{E571G} prp2::hphMX pRS415-Prp2^{WT}	Copper sensitive strain expressing Hsh155^{E571G} and Prp2^{WT} from plasmids. Used for temperature assays.
yAAH2217	MATα cup1Δ ura3 his3 trp1 lys2 ade2 leu2 Hsh155::KanMx, pRS414-Hsh155^{E571K} prp2::hphMX pRS415-Prp2^{WT}	Copper sensitive strain expressing Hsh155^{E571K}and Prp2^{WT} from plasmids. Used for temperature assays.
yAAH2220	MATα cup1Δ ura3 his3 trp1 lys2 ade2 leu2 Hsh155::KanMx, pRS414-Hsh155^{I626Q} prp2::hphMX pRS415-Prp2^{WT}	Copper sensitive strain expressing Hsh155^{I626Q} and Prp2^{WT} from plasmids. Used for temperature assays.
yAAH1916	MATα cup1Δ ura3 his3 trp1 lys2 ade2 leu2 Hsh155::KanMx, pRS414-Hsh155^{WT} prp2::hphMX pRS415-Prp2^{Q548N}	Copper sensitive strain expressing Hsh155^{WT} and Prp2^{Q584N} from plasmids. Used for temperature assays.
yAAH2224	MATα cup1Δ ura3 his3 trp1 lys2 ade2 leu2 Hsh155::KanMx, pRS414-Hsh155^{V502F} prp2::hphMX pRS415-Prp2^{Q548N}	Copper sensitive strain expressing Hsh155^{V502F} and Prp2^{Q584N} from plasmids. Used for temperature assays.
yAAH2223	MATα cup1Δ ura3 his3 trp1 lys2 ade2 leu2 Hsh155::KanMx, pRS414-Hsh155^{E529K} prp2::hphMX pRS415-Prp2^{Q548N}	Copper sensitive strain expressing Hsh155^{E529K} and Prp2^{Q584N} from plasmids. Used for temperature assays.
yAAH2226	MATα cup1Δ ura3 his3 trp1 lys2 ade2 leu2 Hsh155::KanMx, pRS414-Hsh155^{E531K} prp2::hphMX pRS415-Prp2^{Q548N}	Copper sensitive strain expressing Hsh155^{E531K} and Prp2^{Q584N} from plasmids. Used for temperature assays.
yAAH1963	MATα cup1Δ ura3 his3 trp1 lys2 ade2 leu2 Hsh155::KanMx, pRS414-Hsh155^{D563G} prp2::hphMX pRS415-Prp2^{Q548N}	Copper sensitive strain expressing Hsh155^{D563G} and Prp2^{Q584N} from plasmids. Used for temperature assays.
yAAH2227	MATα cup1Δ ura3 his3 trp1 lys2 ade2 leu2 Hsh155::KanMx, pRS416-Hsh155^{E571G} prp2::hphMX pRS415-Prp2^{Q548N}	Copper sensitive strain expressing Hsh155^{E571G} and Prp2^{Q584N} from plasmids. Used for temperature assays.
yAAH2228	MATα cup1Δ ura3 his3 trp1 lys2 ade2 leu2 Hsh155::KanMx, pRS416-Hsh155^{E571K} prp2::hphMX pRS415-Prp2^{Q548N}	Copper sensitive strain expressing Hsh155^{E571K} and Prp2^{Q584N} from plasmids. Used for temperature assays.
yAAH2225	MATα cup1Δ ura3 his3 trp1 lys2 ade2 leu2 Hsh155::KanMx, pRS416-Hsh155^{I626Q} prp2::hphMX pRS415-Prp2^{Q548N}	Copper sensitive strain expressing Hsh155^{I626Q} and Prp2^{Q584N} from plasmids. Used for temperature assays.
Y2H GOLD	MATa, trp1-901, leu2-3, 112, ura3-52, his3-200, gal4Δ, gal80Δ, LYS2::GAL1UAS–Gal1TATA–His3, GAL2UAS–Gal2TATA–Ade2 URA3::MEL1UAS–Mel1TATA AUR1-C MEL1	Strain used to test interactions by Y2H (Clontech).

yAAH0595	MATa, trp1-901, leu2-3, 112, ura3-52, his3-200, gal4Δ, gal80Δ, LYS2::GAL1UAS–Gal1TATA–His3, GAL2UAS–Gal2TATA–Ade2 URA3::MEL1UAS–Mel1TATA AUR1-C MEL1	Strain used as negative control for Y2H assay ((AD HSH155^{WT}; BD Empty)
yAAH2329	MATa, trp1-901, leu2-3,112, ura3-52, his3-200,gal4Δ, gal80Δ, LUS2: :GAL1UAS-Gal1TATA-His3, GAL2UAS-Gal2TATA-Ade URA3: :MEL1UAS-Mel1TATA AUR1-C	Strain used to test the interaction between HSH155 WT and PRP5 using Y2H assay
yAAH2330	MATa, trp1-901, leu2-3,112, ura3-52, his3-200,gal4Δ, gal80Δ, LUS2: :GAL1UAS-Gal1TATA-His3, GAL2UAS-Gal2TATA-Ade URA3: :MEL1UAS-Mel1TATA AUR1-C	Strain used as negative control for Y2H assay for probing the interactions against Prp5
yAAH2331	MATa, trp1-901, leu2-3,112, ura3-52, his3-200,gal4Δ, gal80Δ, LUS2: :GAL1UAS-Gal1TATA-His3, GAL2UAS-Gal2TATA-Ade URA3: :MEL1UAS-Mel1TATA AUR1-C	Strain used to test the interaction between the HSH155^{V502F} allele and Prp5 (AD HSH155^{V502F}; BD PRP5)
yAAH2332	MATa, trp1-901, leu2-3,112, ura3-52, his3-200,gal4Δ, gal80Δ, LUS2: :GAL1UAS-Gal1TATA-His3, GAL2UAS-Gal2TATA-Ade URA3: :MEL1UAS-Mel1TATA AUR1-C	Strain used to test the interaction between the HSH155^{D563G} allele and PRP5 (AD HSH155^{D563G} ;BD PRP5)
yAAH2333	MATa, trp1-901, leu2-3,112, ura3-52, his3-200,gal4Δ, gal80Δ, LUS2: :GAL1UAS-Gal1TATA-His3, GAL2UAS-Gal2TATA-Ade URA3: :MEL1UAS-Mel1TATA AUR1-C	Strain used to test the interaction between the HSH155^{E571K} allele and PRP5 (AD HSH155^{E571K} ; BD PRP5)
yAAH2335	MATa, trp1-901, leu2-3,112, ura3-52, his3-200,gal4Δ, gal80Δ, LUS2: :GAL1UAS-Gal1TATA-His3, GAL2UAS-Gal2TATA-Ade URA3: :MEL1UAS-Mel1TATA AUR1-C	Used to generate strains for Y2H assay after transformation with pGADT7 Hsh155 mutant plasmids.
yAAH2336	MATa, trp1-901, leu2-3,112, ura3-52, his3-200,gal4Δ, gal80Δ, LUS2: :GAL1UAS-Gal1TATA-His3, GAL2UAS-Gal2TATA-Ade URA3: :MEL1UAS-Mel1TATA AUR1-C	Strain used to test the interaction between HSH155 WT and PRP3 using Y2H assay (AD HSH155 WT; BD PRP3)
yAAH2337	MATa, trp1-901, leu2-3,112, ura3-52, his3-200,gal4Δ, gal80Δ, LUS2: :GAL1UAS-Gal1TATA-His3, GAL2UAS-Gal2TATA-Ade URA3: :MEL1UAS-Mel1TATA AUR1-C	Strain used as negative control for Y2H assay for probing the interactions against Prp5 (AD Empty; BD PRP3)
yAAH2338	MATa, trp1-901, leu2-3,112, ura3-52, his3-200,gal4Δ, gal80Δ, LUS2: :GAL1UAS-Gal1TATA-His3, GAL2UAS-Gal2TATA-Ade URA3: :MEL1UAS-Mel1TATA AUR1-C	Strain used to test the interaction between the HSH155^{V502F} allele and Prp5 (AD HSH155^{V502F}; BD PRP5)
yAAH2339	MATa, trp1-901, leu2-3,112, ura3-52, his3-200,gal4Δ, gal80Δ, LUS2: :GAL1UAS-Gal1TATA-His3, GAL2UAS-Gal2TATA-Ade URA3: :MEL1UAS-Mel1TATA AUR1-C	Strain used to test the interaction between the HSH155^{D563G} allele and PRP5 (AD HSH155^{D563G} ;BD PRP5)
yAAH2340	MATa, trp1-901, leu2-3,112, ura3-52, his3-200,gal4Δ, gal80Δ, LUS2: :GAL1UAS-Gal1TATA-His3, GAL2UAS-Gal2TATA-Ade URA3: :MEL1UAS-Mel1TATA AUR1-C	Strain used to test the interaction between the HSH155^{E571K} allele and PRP5 (AD HSH155^{E571K} ; BD PRP5)
