# Supplementary material for: Impact of cancer-associated mutations in Hsh155/SF3b1 HEAT repeats 9-12 on pre-mRNA splicing in Saccharomyces cerevisiae
Source: PLoS One. 2020 Apr 22;15(4):e0229315. doi: 10.1371/journal.pone.0229315 (PMC7176370; doi:10.1371/journal.pone.0229315)
Supplement: S2 Table — (PDF) [file pone.0229315.s003.pdf]

S2 Table. Plasmid used in this study.

Plasmid ID	Plasmid Name	Description
pAAH0403	pRS414-Hsh155^{WT}	Plasmid used to generate Hsh155^{WT}
pAAH1077	pRS414-Hsh155^{V502F}	Plasmid used to generate Hsh155^{V502F} strain. Cloned using SDM of pAAH0403
pAAH1053	pRS414-Hsh155^{E529K}	Plasmid used to generate Hsh155^{E529K} strain. Cloned using SDM of pAAH0403
pAAH1078	pRS414-Hsh155^{E531K}	Plasmid used to generate Hsh155^{E531K} strain. Cloned using SDM of pAAH0403
pAAH1037	pRS414-Hsh155^{D563G}	Plasmid used to generate Hsh155^{D563G} strain. Cloned using SDM of pAAH0403
pAAH1075	pRS414-Hsh155^{E571G}	Plasmid used to generate Hsh155^{E571G} strain. Cloned using SDM of pAAH0403
pAAH1076	pRS414-Hsh155^{E571K}	Plasmid used to generate Hsh155^{E571K} strain. Cloned using SDM of pAAH0403
pAAH1079	pRS414-Hsh155^{I626Q}	Plasmid used to generate Hsh155^{I626Q} strain. Cloned using SDM of pAAH0403
pAAH0404	pRS414-Hsh155^{D540G}	Plasmid used to generate Hsh155^{D540G} strain. Cloned using SDM of pAAH0403
pAAH0405	pRS414-Hsh155^{H331D}	Plasmid used to generate Hsh155^{H331D} strain. Cloned using SDM of pAAH0403
pAAH0818	pRS414-Hsh155^{K740R}	Plasmid used to generate Hsh155^{K740R} strain. Cloned using SDM of pAAH0403
pAAH0820	pRS414-Hsh155^{N747A}	Plasmid used to generate Hsh155^{N747A} strain. Cloned using SDM of pAAH0403
pAAH1098	pRS414-Hsh155$^{H331D/V502F}$	Plasmid used to generate Hsh155$^{H331D/V502F}$ strain. Cloned using SDM of pAAH0403
pAAH1099	pRS414-Hsh155$^{D540G/V502F}$	Plasmid used to generate Hsh155$^{D540G/V502F}$ strain. Cloned using SDM of pAAH0403
pAAH1100	pRS414-Hsh155$^{K740R/V502F}$	Plasmid used to generate Hsh155$^{K740R/V502F}$ strain. Cloned using SDM of pAAH0403
pAAH1101	pRS414-Hsh155$^{N747A/V502F}$	Plasmid used to generate Hsh155$^{N747A/V502F}$ strain. Cloned using SDM of pAAH0403
pAAH0470	ACT1CUP1 WT	WT reporter used for ACT1CUP1 assays.
pAAH0524	ACTCUP1 A258U	BS A258U mutant reporter used for ACT1CUP1 assays.
pAAH0439	ACTCUP1 U257C	BS U257C mutant reporter used for ACT1CUP1 assays.
pAAH0880	ACTCUP1 BS G	BS A259G mutant reporter used for ACT1CUP1 assays.
	pRS415-Prp2^{WT}	Plasmid used to generate Prp^{WT} strains.
pAAH0790	pRS415-Prp2^{Q548N}	Plasmid used to generate Prp2^{Q548N} strains. Cloned using SDM of pRS415-Prp2
pAAH0499	pGADT7-Hsh155^{WT}	Plasmid used to generate pGADT7-Hsh155 mutants.
pAAH0485	pGADT7-Empty	Plasmid used to generate control strains for Y2H assay.
pAAH1149	pGADT7-Hsh155^{V502F}	Plasmid used to generate Prp5 vs Hsh155^{V502F} and Prp3 vs Hsh155^{V502F} strains for Y2H assay. Cloned using SDM of pAAH0499
pAAH1150	pGADT7-Hsh155^{D563G}	Plasmid used to generate Prp5 vs Hsh155^{D563G} and Prp3 vs Hsh155^{D563G} strains for Y2H assay. Cloned using SDM of pAAH0499
pAAH1151	pGADT7-Hsh155^{E571K}	Plasmid used to generate Prp5 vs Hsh155^{E571K} and Prp3 vs Hsh155^{E571K} strains for Y2H assay. Cloned using SDM of pAAH0499
pAAH1153	pGBKT7-Prp3	Plasmid used to generate Prp3 strains for Y2H assay. Gene insert using
